# Measuring What Matters to Individuals with Angelman Syndrome and Their Families: Development of a Patient-Centered Disease Concept Model

**DOI:** 10.1007/s10578-020-01051-z

**Published:** 2020-09-02

**Authors:** Tom Willgoss, Daiana Cassater, Siobhan Connor, Michelle L. Krishnan, Meghan T. Miller, Carla Dias-Barbosa, Dawn Phillips, Julie McCormack, Lynne M. Bird, Rebecca D. Burdine, Sharon Claridge, Terry Jo Bichell

**Affiliations:** 1 Roche Products Limited, Hexagon Place, 6 Falcon Way, Shire Park, Welwyn Garden City, AL7 1TW UK; 2 Roche Innovation Center Basel, F. Hoffmann-La Roche Ltd, Basel, Switzerland; 3 Evidera, Hammersmith, London, UK; 4 Division of Physical Therapy, School of Medicine, University of North Carolina, Chapel Hill, NC USA; 5 Evidera, Patient-Centered Research, Bethesda, MD USA; 6 Department of Pediatrics, University of California San Diego, San Diego, CA USA; 7 Department of Molecular Biology, Princeton University, Princeton, NJ USA; 8 Foundation for Angelman Syndrome Research (FAST), Downers Grove, IL USA; 9 Consortium for Outcome Measures and Biomarkers for Neurodevelopmental Disorders, Nashville, TN USA

**Keywords:** Angelman syndrome, Qualitative research, Patient-centered, Outcome assessment, Clinical endpoint

## Abstract

Angelman syndrome (AS) is a complex, heterogeneous, and life-long neurodevelopmental disorder. Despite the considerable impact on individuals and caregivers, no disease-modifying treatments are available. To support holistic clinical management and the development of AS-specific outcome measures for clinical studies, we conducted primary and secondary research identifying the impact of symptoms on individuals with AS and their unmet need. This qualitative research adopted a rigorous step-wise approach, aggregating information from published literature, then evaluating it via disease concept elicitation interviews with clinical experts and caregivers. We found that the AS-defining concepts most relevant for treatment included: impaired expressive communication, seizures, maladaptive behavior, cognitive impairment, motor function difficulties, sleep disturbance, and limited self-care abilities. We highlight the relevance of age in experiencing these key AS concepts, and the difference between the perceptions of clinicians and caregivers towards the syndrome. Finally, we outline the impact of AS on individuals, caregivers, and families.

## Introduction

Angelman syndrome (AS) is a neurodevelopmental disorder resulting from deficient expression or function of the maternally expressed *UBE3A* gene [1]. This can be due to one of four mechanisms: chromosome 15q11q13 deletion encompassing the *UBE3A* gene, intragenic *UBE3A* mutation, paternal uniparental disomy (UPD) for chromosome 15, or an imprinting defect (ID) [2]. Population estimates report the prevalence of AS to be between 1 in 10,000 and 1 in 20,000 [3–5].

The developmental trajectory of individuals with AS begins to deviate from that of the typically developing population in the first months of life; however, AS is usually diagnosed after the first year of age, when the overall clinical characteristics start to become apparent [6]. Individuals with AS present with global developmental delay that persists, resulting in functionally severe cognitive impairment, behavioral difficulties, and physical difficulties, which cause high caregiver burden and need for societal support [7]. AS symptoms are both complex and heterogeneous in presentation. According to the consensus diagnostic criteria for AS, the classic symptoms can be categorized as consistent, frequent, or associated features—depending on their likelihood of presentation [8]. The ‘consistent features’ are the core clinical elements of AS that comprise developmental delay, movement disorder, and speech impairment; these are seen in essentially all people diagnosed with AS. The ‘frequent features’ are reported in more than 80% of individuals with AS and include seizures and abnormal EEG. The ‘associated features’ are often part of the overall clinical phenotype for AS but occur in 20–80% of individuals with AS, including disturbed sleep, and excessive chewing or frequent drooling. While all these clinical features are important for characterizing AS, they are not expected to be equally important to clinicians for clinical management, or to caregivers for quality of life impacts.

Despite being a reasonably well-characterized, complex, and severe condition, there is a paucity of literature reporting the impact of AS on individuals and their families. For example, studies have found that daily management of seizures and disturbances of sleep in the AS individual are significant contributors to associated stress and sleep deficits, which can lead to clinically significant anxiety and depression in family members. Among parents of people with AS, up to 71% of mothers and 42% of fathers report clinical levels of anxiety, while 21% of mothers and 33% of fathers report clinical levels of depression [7].

Currently there are no approved treatments specifically for AS, with standard care aimed at symptomatic relief through pharmacological and non-pharmacological approaches [9]. Given the complexity of AS and the lack of targeted treatment options, clinical management can require the involvement of several specialists and can vary significantly between individual patients and between different countries [10]. A better understanding is needed of the key elements of AS and the impact of these elements on individuals and their families over their lifespan, to identify the areas of highest unmet need which warrant treatment. This insight can support the implementation of effective care coordination, to ensure that individuals with AS achieve the best outcomes possible [11]. An improved understanding of the disease-defining concepts of AS, and those concepts that would be most beneficial to treat, would support more comprehensive, coordinated, and holistic clinical management for individuals with AS and their families [12].

Several potential future therapies for AS are being developed, targeting a variety of symptoms using diverse disease-modifying approaches, including strategies to modify *UBE3A* expression [9]. In order to accurately interpret the clinical meaning of efficacy of an intervention within a clinical trial, there is the need to accurately measure patient-centered treatment effect built on a rigorous, research-based, and patient-centered description of the disease-defining concepts of AS and those aspects that are most important for treatment [13, 14].

Here we report a patient-centered conceptual model of AS, including rating of the concepts important for treatment, and the age-relevance of key concepts. In addition to improving coordinated clinical care, this research also supports the development of future AS-specific clinical outcomes assessments (COAs) because the selection of outcome measures for clinical trials should be underpinned by an understanding of which disease concepts are most relevant to individuals, and which concepts should be the focus for new therapy development. This patient-centric principle is at the heart of the Food and Drug Administration’s (FDA) guidance [14], which recommends that qualitative research be undertaken to identify concepts of importance to individuals, and that a conceptual model of a condition be developed to illustrate how signs, symptoms, and impacts interact, revealing which are important to treat. To complement this, we additionally sought to further document the daily experience of caring for and supporting an individual with AS from the caregiver’s perspective. This can also provide important insights to healthcare professionals working with patients with AS to improve their understanding of both the individual’s and their caregivers’ needs and preferences.

## Methods

We utilized a step-wise approach in line with FDA guidance, starting with identifying concepts of interest in AS from the published literature, through to finalizing the AS conceptual model and treatment needs based on the feedback from caregivers and expert clinicians [14].

### Literature Review

A targeted review of published articles was conducted to characterize AS signs and symptoms and their impacts on individuals and caregivers; to draft an AS conceptual model, and to inform the development of the clinician and caregiver interview discussion guides. The search was conducted in PubMed and included, publications between January 1, 2013 and July 24, 2017 with a population of individuals and caregivers of individuals with AS. The following study designs were included: clinical trials, interventional studies, observational studies, meeting and conference abstracts, letters, news, comments, editorials, and case reports. There were no language restrictions.

A draft conceptual model of AS was developed based on this literature review, and then revised and clarified through qualitative interviews of clinical experts and caregivers of people with AS.

The literature review also informed the semi-structured interview guides developed to facilitate open discussion with the clinicians and caregivers. The interview guides were reviewed by representatives of the Angelman Biomarkers and Outcome Measures (A-BOM) Alliance, a pre-competitive alliance of researchers, clinicians, industry, and patient advocates with a mission to identify sensitive, specific, and patient-centered biomarkers and outcome measures for AS [15].

### Clinical Expert Interviews

In-depth, telephone-based, concept elicitation interviews were conducted with three clinical experts who specialize in the treatment of AS, have a good understanding of global research in AS, and who also regularly follow patients with other cognitive and motor disabilities. To incorporate different clinical practices and cultural expectations, we included clinicians from the USA and the Netherlands. These countries are also sites of specialist AS research centers, ensuring that clinical experts had an excellent understanding of the disease. All clinical experts were recruited in collaboration with the A-BOM Alliance and reimbursed for their time at fair market value (FMV) rates. The FMV rate was determined based on remuneration that values the time of a healthcare professional with a particular skill set in a particular market and did not exceed USD 290 per hour. The objectives of the interviews were to: inform the development of the caregiver interview guide; better understand the clinical manifestations and natural history of AS; evaluate the impact of AS on individual’s and caregivers’ daily lives; discuss the symptoms experienced by individuals with AS at different ages and with different genetic variants; understand how caregivers’ lives are impacted by caring for a person with AS and which aspects of AS are the most challenging for caregivers to manage in the short- and long-term; report the meaningful treatment outcomes from a clinical perspective; and provide feedback on the draft conceptual model with a particular focus on any missing concepts.

All interviews were audio recorded and transcribed *verbatim*. Transcripts were reviewed and key content relating to symptoms and impacts were incorporated into the conceptual model.

### Caregiver Interviews

In-depth, telephone-based, concept elicitation interviews were also conducted with 30 caregivers of individuals with AS. These caregiver interviews provided a complimentary perspective to the clinical experts. The objectives of the interviews were to: obtain insights about the course of AS and the impact on patients’ and caregivers’ daily lives; identify which life domains are most impacted and which concepts are most important to individuals and caregivers; understand treatment pathways and healthcare resources used; and collect caregivers’ expectations regarding new treatments, as well as identifying preferences for attributes of treatment and features of AS to be preferentially targeted by future therapies.

The caregiver interview guide was updated to incorporate feedback from the clinical expert interviews. Clinicians and caregivers were recruited from the USA and the Netherlands to reflect different clinical practices and cultural expectations in both North America and Europe. Caregivers were required to meet the following criteria: 18 years of age or older; the parent or guardian of an individual with AS who is aged 2 years of age or older and who has an AS diagnosis confirmed by genetic testing, and living with the AS individual. Caregivers were recruited through advocacy groups in the USA and the Netherlands, including the Angelman Syndrome Foundation (ASF), Foundation for Angelman Syndrome Therapeutics (FAST), and the Nina Foundation, using Institutional Review Board (IRB)-approved recruitment materials. All consent and study procedures were approved by Ethical and Independent Review Services, an IRB based in Independence, Missouri, USA, and Medische Ethische Toetsings Commissie Erasmus MC (IRB) based in Rotterdam, the Netherlands. The study was approved by Ethical and Independent Review Services on February 6, 2018 (study number 18000-01); Medische Ethische Toetsings Commisie Erasmus MC approved the protocol for use with the Dutch population on April 12, 2018 (study number MEC-2018-084). Efforts were made to recruit caregivers of individuals with AS from different categories of: age (e.g., 2–4 years, 5–8 years, 9–12 years, 13–18 years, and over 18 years), sex, race/ethnicity, genotype, and range of functional levels (i.e., ambulatory vs. wheelchair dependent). These categories were pre-specified to ensure recruitment was representative of AS, but these would not be sufficient to support sub-group analysis. Interviews were conducted between March 2, 2018 and June 11, 2018.

Interviews were conducted in the caregiver’s native language. Caregivers were asked about the symptoms experienced by the individuals they cared for, as well as the impact of these symptoms on both the person with AS and on themselves as caregivers. Interviews were transcribed *verbatim* and translated into English if conducted in Dutch. A content analysis approach was undertaken to analyze the data (based on notes, transcripts, and audio recordings) from the qualitative interviews using ATLAS.ti (version 7.5) qualitative data analysis software. A coding dictionary was developed based on the themes and concepts that emerged after the first three interviews.

**Fig. 1:**
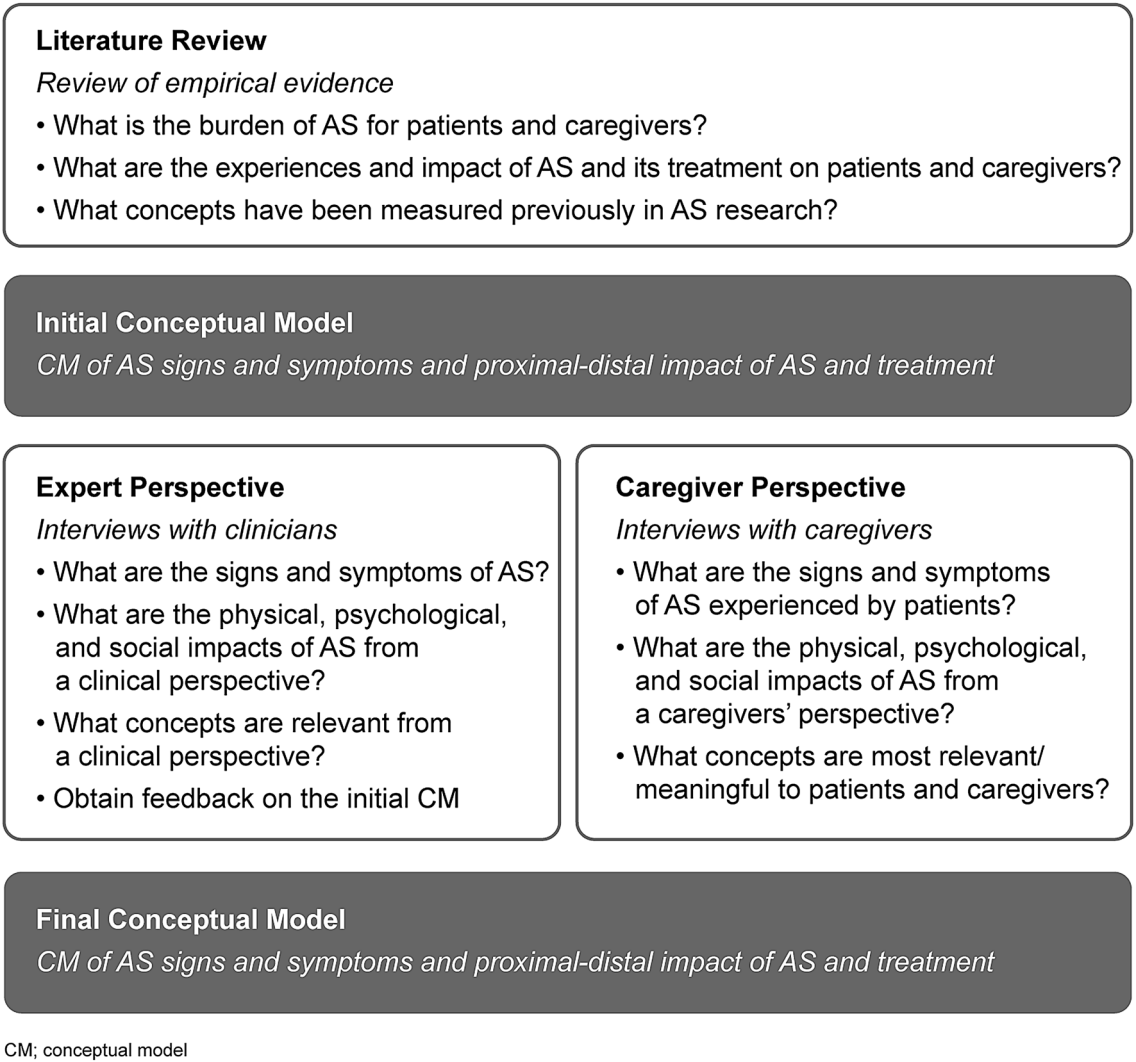
Stepwise approach to developing the AS disease concept model

The FDA requests evidence of ‘saturation’ in qualitative research to ensure that all content relevant to the target sample are captured [14]. Therefore, a saturation grid was developed to establish and document conceptual saturation for the caregiver interviews. Conceptual saturation was defined, a priori, as the point at which no substantially new themes, descriptions of a concept, or terms are introduced as additional interviews are conducted [16]. Of note, conceptual saturation was not explored for the clinical expert interviews, as the primary goal was not concept elicitation, but to develop the caregiver interview guide, solicit feedback on the draft conceptual model and better understand the natural history of disease. A schematic summarizing the methodological approach taken is presented below (Fig. 1).

## Results

### Literature Review and the Draft Conceptual Model

Twenty articles were reviewed as a preliminary step to characterizing AS signs, symptoms, and impacts. Following a review of the scope and content of these 20 articles, only seven were selected for data extraction. The remaining 13 articles were excluded due to limitations of the study design (e.g., case reports) and/or a lack of relevant information to inform the draft AS conceptual model. Reviewed articles included: an updated consensus for AS diagnostic criteria, published AS Clinical Management Guidelines, and a conceptual model of AS published as a conference abstract [8, 17, 18].

This literature review reported key AS symptoms related to neurological, musculoskeletal, gastrointestinal, dermatological, and visual systems, as well as impacts on activities and behaviors, including development and cognition, communication, sleep, gait and balance, behavior, and self-care [8, 9, 19–22].

In addition to symptoms and impacts associated with AS, the literature review also explored the effects of AS on caregivers. Caregivers reported not having the time or the energy to take care of themselves, and also experienced additional stress when attempting to arrange care for the AS individual in order to attend to personal matters [23].

A draft conceptual model of AS was developed based on data from the literature review. Concepts were organized into three overarching domains, based on the World Health Organizations International Classification of Functioning (WHO ICF) [24]: ‘Disease Defining Concepts’, ‘Individual Impact Concepts’, and ‘Caregiver Impact Concepts and Modifying Factors’. This draft model included AS-defining concepts, proximal impact concepts and distal impact concepts. AS-defining concepts were defined as: Neurological; Musculoskeletal; Gastrointestinal; Skin; and Visual domains. Proximal impact concepts included: Cognition; Motor; Communication; Behavior; Sleep; and Self-care domains. Distal impact concepts included: Community/School; Socialization and Family Life; Caregiver Burden; and Modifying Factors. This initial draft conceptual model was reviewed during qualitative interviews with clinical experts and caregivers, and then refined based on their feedback.

### Clinical Expert Feedback and Link to AS Genotype

Three specialists with extensive experience treating people with AS participated in these concept elicitation interviews; two from the USA and one from the Netherlands. These clinical experts had each seen between 60 and 105 individuals with AS ranging from 1 to 40 years of age.

All three clinical experts agreed that there is no typical presentation of AS, but that many individuals share similar characteristics, including: delayed or decreased communication (e.g., the inability to produce any words or very few words spoken); ataxia, or other motor challenges; and significant cognitive disabilities. In addition, clinicians mentioned seizures, disturbed sleep, and disruptive behavior.

These aspects of AS were included in the draft conceptual model developed from the pragmatic literature review, so no domains were found to be missing. The expert clinician interviews provided important insights from their breadth of clinical experience across patient ages, across two cultures, and from the 250 individuals with AS collectively treated by them. These interviews provided descriptions of how symptoms present in people with AS, and their impact on individuals and their caregivers. The interview guide for caregivers was updated to incorporate this expert clinical feedback.

All three clinical experts agreed there are differences in AS symptom presentation linked to the individual’s genotype. All clinicians confirmed that individuals with deletion had the most severe symptoms and tended to be the most severely affected, while individuals with *UBE3A* mutations, UPD or ID tended to be less impacted. Two of the three clinicians reported that individuals with *UBE3A* mutations have better communication skills than individuals with deletion-type AS. All experts agreed that AS individuals had a similar clinical presentation regardless of their sex.

### Caregiver Feedback and the Impact of AS on Individuals and Their Families

#### Sociodemographic Characteristics

The sociodemographic characteristics of the 30 caregivers were comparable between the USA and the Netherlands, where 25 and five caregivers were recruited, respectively (Table 1). The majority of the caregivers were female (n = 28; 93%), of White ethnicity (n = 25, 83%), married (n = 25, 83%), and in full-time or part-time employment. The mean caregiver age was 44 ± 9 years old. Overall, the caregivers were well educated, with over half holding a university or college degree or a postgraduate degree.

**Table 1:** Sociodemographic characteristics of caregivers

Characteristic of caregiver	USA (n = 25)	Netherlands (n = 5)	Total (N = 30)
Age (years)			
Mean (SD)	44.6 (9.8)	42.8 (2.6)	44.3 (9.0)
Median (range)	45.0 [26.0–61.0]	42.0 [40.0–47.0]	43.0 [26.0–61.0]
Relationship to individual with AS, n (%)			
Mother/step-mother	23 (92.0%)	5 (100.0%)	28 (93.3%)
Father/step-father	2 (8.0%)	0 (0.0%)	2 (6.7%)
Ethnicity, n (%)^a,b^			
Hispanic or Latino	2 (8.0%)	0 (0.0%)	2 (8.0%)
Not Hispanic or Latino	22 (88.0%)	0 (0.0%)	22 (88.0%)
Missing	1 (4.0%)	0 (0.0%)	1 (4.0%)
Racial background, n (%)			
White	20 (80.0%)	5 (100.0%)	25 (83.3%)
Asian	2 (8.0%)	0 (0.0%)	2 (6.7%)
Native Hawaiian or other Pacific Islander	1 (4.0%)	0 (0.0%)	1 (3.3%)
American Indian or Alaska Native	1 (4.0%)	0 (0.0%)	1 (3.3%)
Other^c^	1 (4.0%)	0 (0.0%)	1 (3.3%)
Marital status, n (%)			
Married	22 (88.0%)	3 (60.0%)	25 (83.3%)
Divorced/separated	2 (8.0%)	0 (0.0%)	2 (6.7%)
Single, in a relationship	1 (4.0%)	1 (20.0%)	2 (6.7%)
Other^d^	0 (0.0%)	1 (20.0%)	1 (3.3%)
Employment status, n (%)			
Employed, full-time or part-time	20 (80.0%)	3 (60.0%)	23 (76.7%)
Homemaker	5 (20.0%)	1 (20.0%)	6 (20.0%)
Disabled	0 (0.0%)	1 (20.0%)	1 (3.3%)
Education status, n (%)			
Secondary/high school	1 (4.0%)	0 (0.0%)	1 (3.3%)
Associate degree, vocational, technical or trade school	1 (4.0%)	1 (20.0%)	2 (6.7%)
Some college (< 1 year)	2 (8.0%)	0 (0.0%)	2 (6.7%)
Some college (2–3 years)	5 (20.0%)	0 (0.0%)	5 (16.7%)
University/college degree	8 (32.0%)	2 (40.0%)	10 (33.3%)
Postgraduate degree	8 (32.0%)	1 (20.0%)	9 (30.0%)
Other education^e^	0 (0.0%)	1 (20.0%)	1 (3.3%)

*SD* standard deviation

^a^USA only

^b^One participant did not answer this question

^c^Other race includes ‘White and Indian’ (n = 1)

^d^Other marital status includes ‘living together’ (n = 1)

^e^Other education includes Middelbaar Beroeps Diploma (n = 1)

The sociodemographic characteristics of the people with AS were comparable between the USA and the Netherlands (Table 2). Just over half the individuals with AS were female (n = 17; 57%). Their mean age was 12 ± 7 years old and their median age was 11 (range between 2 and 29) years old; six were individuals between 18 and 29 years of age. Most of the individuals with AS were of White ethnicity (n = 23, 76%) and had attended school (n = 29; 97%). The majority of caregivers reported that the individual they cared for had very good or excellent overall health (n = 17; 57%). Sixty percent of individuals with AS had chromosomal deletion and the remaining 40% included individuals with *UBE3A* mutation, UPD, and ID.

**Table 2:** Sociodemographic characteristics of the individuals with AS

Characteristic of individual with AS	USA (N = 25)	Netherlands (N = 5)	Total (N = 30)
Age (years)			
Mean (SD)	13.0 (7.2)	9.4 (3.6)	12.4 (6.8)
Median (range)	12.0 [2.0–29.0]	8.0 [6.0–15.0]	11.0 [2.0–29.0]
Sex, n (%)			
Male	13 (52.0%)	0 (0.0%)	13 (43.3%)
Female	12 (48.0%)	5 (100.0%)	17 (56.7%)
Ethnicity, n (%)^a,b^			
Hispanic or Latino	3 (12.0%)	0 (0.0%)	3 (12.0%)
Not Hispanic or Latino	21 (84.0%)	0 (0.0%)	21 (84.0%)
Racial background, n (%)^a,c,d^			
White	19 (76.0%)	4 (80.0%)	23 (76.7%)
Native Hawaiian or other Pacific Islander	1 (4.0%)		1 (4.0%)
American Indian or Alaska Native	1 (4.0%)		1 (4.0%)
Multiple/mixed races		1 (20.0%)	1 (20.0%)
Other^b^	4 (16.0%)		4 (16.0%)
Attended school, n (%)			
Yes	24 (96.0%)	5 (100.0%)	29 (96.7%)
No	1 (4.0%)	0 (0.0%)	1 (3.3%)
Type of school attended, n (%)			
No schooling outside of home	1 (4.0%)	0 (0.0%)	1 (3.3%)
Pre-school	3 (12.0%)	0 (0.0%)	3 (10.0%)
Grade 2	1 (4.0%)	1 (20.0%)	2 (6.7%)
Grade 3	1 (4.0%)	0 (0.0%)	1 (3.3%)
Grade 4	1 (4.0%)	1 (20.0%)	2 (6.7%)
Grade 5	3 (12.0%)	0 (0.0%)	3 (10.0%)
Grade 6	0 (0.0%)	1 (20.0%)	1 (3.3%)
Grade 7	2 (8.0%)	0 (0.0%)	2 (6.7%)
Grade 8	1 (4.0%)	0 (0.0%)	1 (3.3%)
Grade 10	2 (8.0%)	0 (0.0%)	2 (6.7%)
Grade 11	1 (4.0%)	0 (0.0%)	1 (3.3%)
Grade 12	1 (4.0%)	0 (0.0%)	1 (3.3%)
Attended school until age 21/aged out of school	3 (12.0%)	0 (0.0%)	3 (10.0%)
Other school^e^	5 (20.0%)	2 (40.0%)	6 (23.3%)
Caregiver during the day, n (%)^f^			
Caregiver participant	18 (72.0%)	5 (100.0%)	23 (76.7%)
Spouse or co-caregiver	10 (40.0%)	1 (20.0%)	11 (36.7%)
Extended family member (e.g., child’s grandparents, aunts, uncles)	4 (16.0%)	2 (40.0%)	6 (20.0%)
Employed childcare	1 (4.0%)	1 (20.0%)	2 (6.7%)
In-home healthcare provider	2 (8.0%)	0 (0.0%)	2 (6.7%)
Daycare	3 (12.0%)	0 (0.0%)	3 (10.0%)
Other^g^	7 (28.0%)	0 (0.0%)	7 (23.3%)
Genotype, n (%)			
Deletion positive for the AS region on chromosome 15	15 (60.0%)	3 (60.0%)	18 (60.0%)
Mutation of the *UBE3A* gene on chromosome 15	5 (20.0%)	1 (20.0%)	6 (20.0%)
UPD	4 (16.0%)	1 (20.0%)	5 (16.7%)
ID	1 (4.0%)	0 (0.0%)	1 (3.3%)

*SD* standard deviation

^a^USA only

^b^One participant did not answer this question

^c^Other race includes: 'White and Asian' (n = 2), 'White and Black' (n = 1) and 'White and Indian' (n = 1). ^d^Dutch only

^e^Other school includes: 'Special education' (n = 1), Primary/Elementary School' (n = 2), 'High School' (n = 1), 'Road School' (n = 1) and 'Special needs school' (n = 1)

^f^Not mutually exclusive

^g^Other caretakers during the day includes: 'School' (n = 4), 'Day Services and Respite Worker' (n = 1), 'In Alliance Program For People With Disabilities' (n = 1) and 'adult day training program' (n = 1)

### AS Symptoms and Impacts Most Frequently Reported by Caregivers

**Fig. 2:**
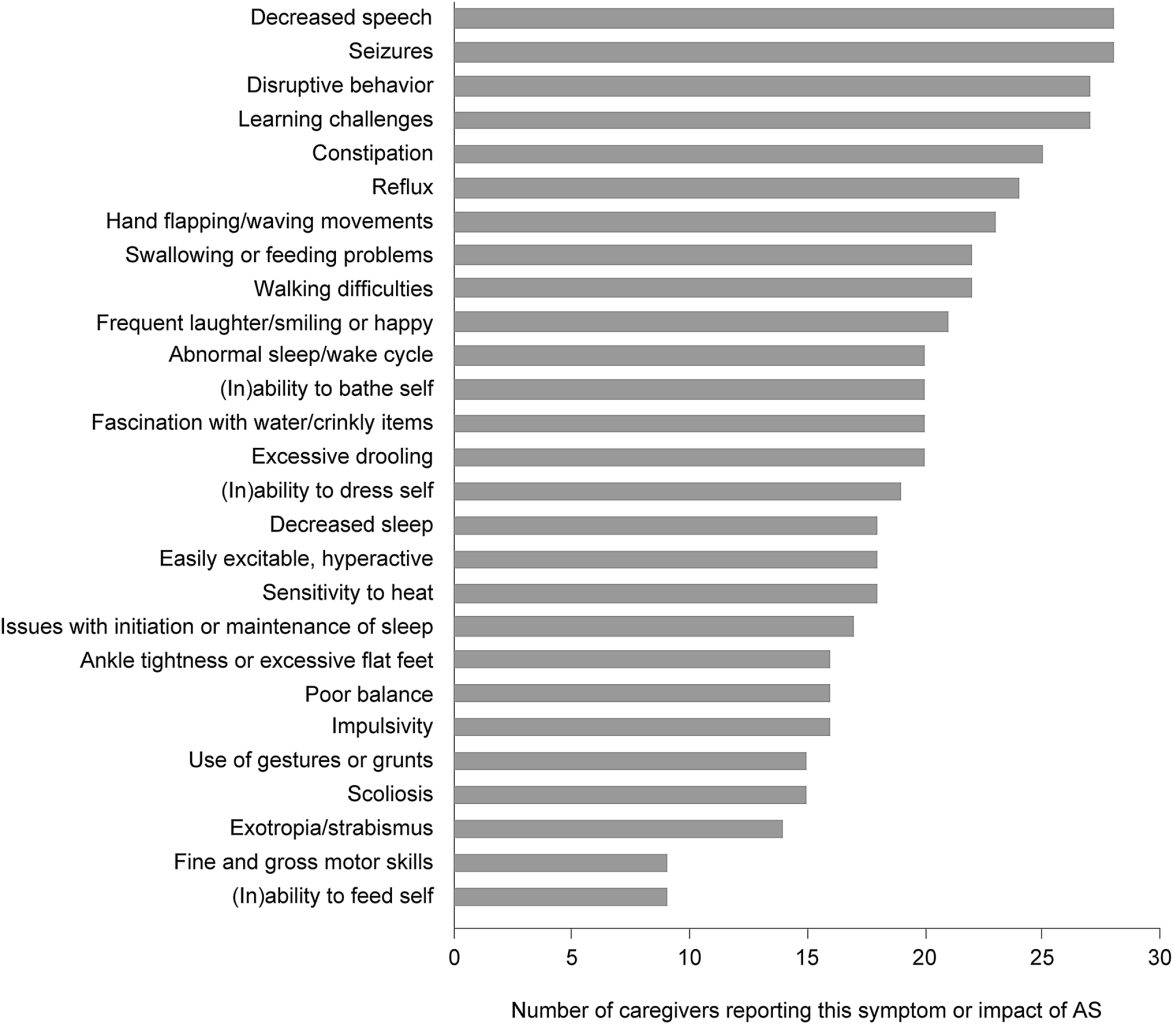
Symptoms and impacts of AS most frequently reported by caregivers

The number of caregivers reporting a specific concept may be linked to how important that concept is for defining a condition. The most frequent concepts reported by AS caregivers were: decreased speech, seizures, disruptive behavior, and learning difficulties (Fig. 2).

### Qualitative Insights into the Key AS Concepts Reported by Caregivers

The in-depth interviews with clinicians and caregivers provided rich insights into key AS concepts, further supported by experiences of living with these AS concepts (Table 3).

**Table 3:** Key AS symptoms and impacts identified by caregivers, with key findings and an example of each from the interview transcripts

Type of AS symptom and key finding	Example from caregiver interviews
Expressive communication impairment is a core component of AS. The majority of caregivers reported that the AS individual they cared for was non-verbal (83%), few AS individuals used 10–20 words (10%). Half the AS individuals used gestures and/or vocalizations to communicate (53%), and a minority of caregivers reporting an AS individual using sign language (27%)	Um, it kind of just depends on how much experience they have with people who are nonverbal. Um, like our family members who've been around him some or, you know, like his grandmother who babysits and, um, you know, we lived with her for the first, uh, seven years of his life, you know, she–she's like me, and she can just pick up on pretty much anything that he needs or wants or is trying to tell her. Um, but like my sister, who he doesn't see nearly as often, you know, it's definitely much more of a struggle for him to, uh, to communicate with her and–and her with him Mother of 11-year-old boy with *UBE3A* deletion, USA
Seizure frequency ranged from multiple times per day (40%), to a few times per month (13%), or a few times per year (23%). Seizures can impact the ability to leave the home, be a reason for frequent hospital visits, and be distressing for the individual and caregiver	… we start seeing like we call shaking seizure, he goes in air and shook for a second or two. And, it’s at least someone electrocuted him, but he couldn’t move his body, he looked scared and after that, you know, he—he cried Mother of 8 year old boy with *UBE3A* mutation, USA
Disruptive behavior can take different forms, for example, hair pulling, biting, grabbing, and pinching. This was reported across all age groups and all AS genotypes	Those have gotten worse over time… when he gets frustrated it’s usually an escalated thing. Um, usually he’ll just push things away to start with and then it goes down, uh, and it—it doesn’t cease or redirect. At that point, his next step is usually he bites his own palm, his right palm, which is calloused like mad… Um, if that doesn’t work he will pull your hair… all of those are still huge, uh, things that he still does to this day Mother of 18-year-old boy with *UBE3A* deletion, USA
Cognitive and/or learning impairment includes impaired judgement, limited concentration and difficulties focusing, although some caregivers also noted their children have a good memory for people and faces	…. Um, that a sharp knife, if he were to, you know, well, clearly he doesn’t have knives in his hands, but if he—he did as a little boy one time, tried to cut his watermelon. You know, we had been cutting a watermelon and turned for a second and I don’t think he had any clue how he could have hurt himself, none whatsoever. But, yes, those are kind of scary things Mother of a 23-year-old son with UBE3A mutation, USA
Motor difficulties include both gross and fine motor: walking difficulties (73%), poor balance (53%) and fine motor skills or general motor skills issues (23%) and tremors or jerky movements	Well, something that perhaps does give a clearer picture: if she would want to grasp a glass of water, then you have a great chance that first her fingertips touch the glass because she doesn't estimate well, and so then she bumps it instead of picking it up well Mother of a 6-year old daughter with UBE3A deletion, Netherlands
Sleep disturbances often included not sleeping well (80%), but could include snoring and teeth grinding (40%), bed-wetting (20%), and sleep terrors (17%). These sleep disturbances were also related to subsequent mood and disruptive behavior (13%). Sleep disturbances tended to improve with age	For his entire life, his sleep patterns have been in cycles. Uh, everything that [name] is—it lacks and annoys, um, it would be good for a week and then horrific for three weeks. Uh, it—it—anytime anything as far as his sleep has improved, it’s only been temporary. Um, currently he sleeps—he’ll fall asleep about 7:30 or 8:00 in the evening and he’ll sleep until about midnight. He’ll be up for two hours, three hours and then try to do it again until—he has to be awakened at 5:00 for school, to get ready for school Mother of 15-year-old boy with UBE3A deletion, USA
Self-care was reported as impaired by most caregivers including requiring assistance (63%) or inability to dress independently (10%). Assistance with meals was required by a third (30%), with some individuals requiring their food to be cut up (20%)	And then-and-and then the-the putting the clothes on with him, oh, my gosh, it’s a like World War III when I’m brushing his teeth, anything that has to do with his-with him touching his-his body, it’s-it’s like World War III in my house when I’m brushing his teeth it’s a struggle Mother of a 5-year old son with chromosome 15 UPD, USA

### Saturation of Concepts in Caregiver Interviews

Key concepts displayed in Fig. 2 were endorsed by at least half of the 30 caregivers interviewed. Saturation was achieved after ten interviews, meaning no new concepts emerged after the first ten interviews had been completed [25]. For other concepts endorsed by five or more caregivers, saturation was achieved after 20 interviews: incontinence (n = 14); ataxia (n = 9); weight concerns (n = 9); attention and concentration troubles (n = 9); hand tremors/tremors (n = 7); memory difficulties (n = 7); visual impairment (n = 6); sensitivity to light (n = 6); bed wetting (n = 6); weakness (n = 5); gag (n = 5); sleep tremors (n = 5); and anxiety/panic attacks (n = 5).

For much rarer concepts, those reported by less than five caregivers, saturation was not or was only partially achieved in this qualitative research. This supports the adequacy of the interview sample size of 30 interviews for reporting key concepts with AS, but also reflects the heterogeneous and complex nature of AS [8].

### Final AS Conceptual Model

**Fig. 3:**
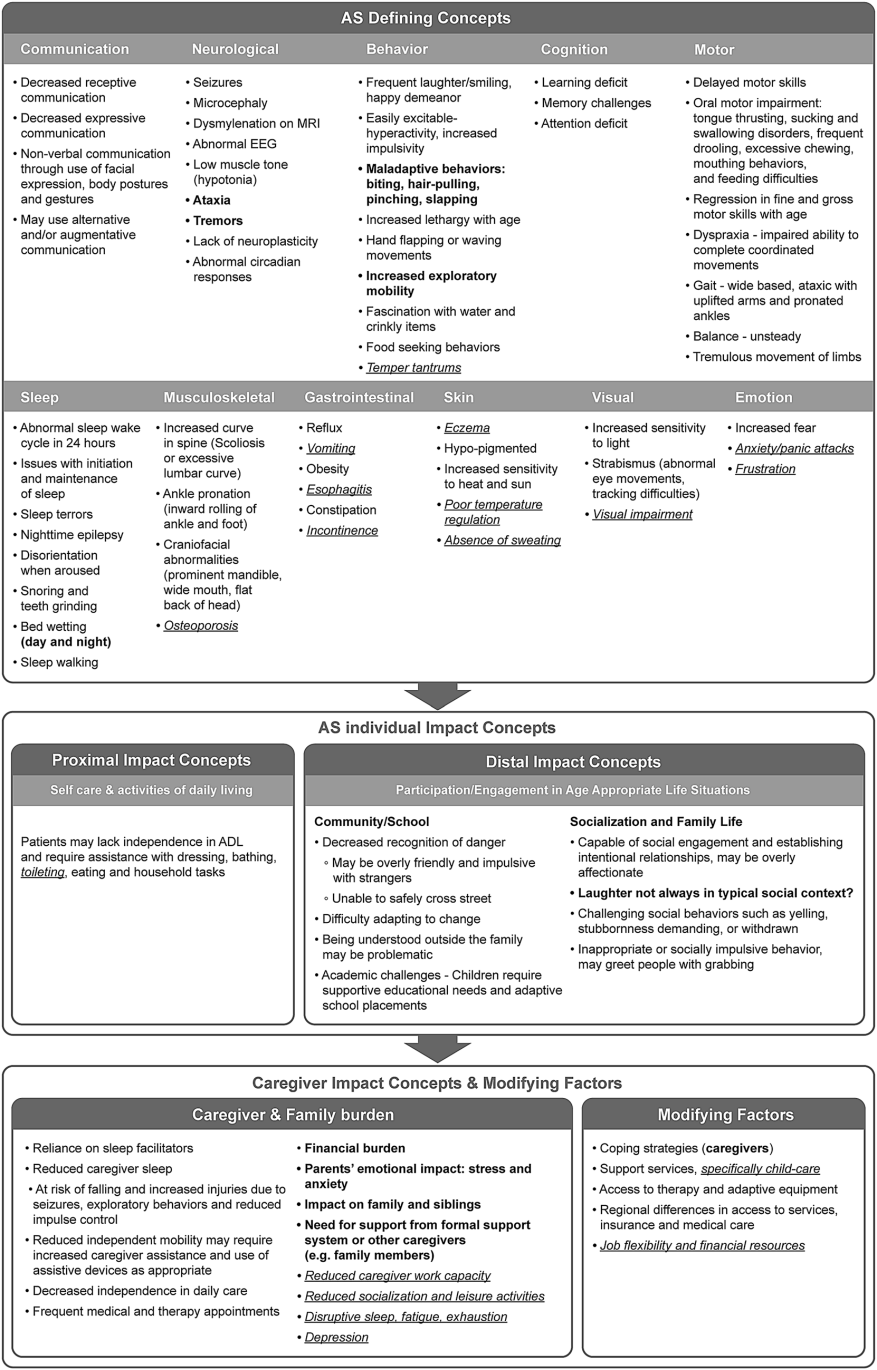
Final conceptual model of AS based on published literature, clinical expert and caregiver concept elicitation feedback

The draft conceptual model, developed from the pragmatic review of published literature, was updated to reflect the clinical expert and caregiver interview feedback gathered (Fig. 3). Concepts continued to be organized into the WHO ICF domains: ‘Disease Defining Concepts’, ‘Proximal and Distal Individual Impact Concepts’, and ‘Caregiver Impact Concepts and Modifying Factors’. Key changes made in this final conceptual model include the re-categorization of more symptoms as being ‘disease defining concepts’, whereas from the literature review alone some symptoms were categorized as being less impactful ‘individual impact concepts’. The concepts finally considered to define AS include: Communication; Neurological; Behavior; Cognition; Motor; Sleep; Musculoskeletal; Gastrointestinal; Skin; Visual; and Emotion. Next are the AS individual impact domains including: Proximal Impact concepts; and Distal Impact concepts. Finally, the caregiver impact concepts and modifying factors include: Caregiver and Family Burden; and Modifying Factors.

In accordance with the iterative nature of this qualitative research, the model shows the history of changes: the draft model concepts from the literature are in black text; clinical expert feedback is in blue text; and caregiver feedback is in red text. This highlights the new information added by this research to the published body of evidence in AS, as well as the differences in perspective between clinicians and caregivers regarding the impacts of AS on individuals and their families.

### Age-Relevance of the Key AS Concepts

All key AS symptoms are present from infancy and childhood, and persist into adulthood, with the exception of obesity and walking difficulties, which are found in adults rather than children [18]. However, symptoms are perceived differently as the person with AS ages through infancy, childhood, adolescence, and adulthood. During concept elicitation interviews, 22 caregivers to people with AS aged between 2 and 29 years old reported the most challenging symptoms they had faced at different ages of their child’s life, and to reflect if these most challenging symptoms had changed over time. There was no limit to the number of challenging symptoms a caregiver could report, so there was variation in reporting rates between caregivers, as well as across age groups.

A detailed description of the changes in AS symptoms and the relative importance of these at different ages are presented in Table 4, with the most challenging symptoms highlighted. Seizures, mobility issues, and sleep problems were considered to be relatively more challenging in young children with AS, but were relatively less challenging as the person grew into adolescence. In contrast, caregivers reported that the challenges associated with impaired expressive communication persisted as the person with AS moved into adolescence. This increased challenge of impaired expressive communication with age could reflect a greater inequality between receptive and expressive communication skills (with increasing frustration) and the persistent challenges faced by people with AS in effectively advocating for themselves in social contexts, a skill which typically becomes more important as individuals age.

**Table 4:** Caregiver-rated most challenging AS symptoms, by age group

Most challenging symptoms	Number of caregivers reporting this AS symptom was challenging, per AS age group		
	≤ 5 years	6–12 years	15–17 years
Communication impairment or decreased speech	**3**	**5**	**4**
Seizures	**5**	2	1
Disruptive behavior	1	**3**	1
Learning challenges	1	1	1
Walking difficulties	**3**	2	1
Sleep issues	**4**	2	1
Ability to use the toilet	1	**3**	1

Bold, underlined numbers show where three or more caregivers reported this AS symptom as being challenging

*AS* Angelman syndrome

Overall, the reasons why the relative impact of AS symptoms change with age are varied, but may include the progressive older age of caregivers, the availability of medications or special care facilities for older individuals in different geographies, or the inherent biological variability of symptom intensity or manifestation. It may also reflect a change in expectations of caregivers over time, or the disparity between the physical maturity of the individual as they grow into adulthood, with the developmental levels of ability. For example, older individuals with AS may be better able to self-soothe during the night and thus may not wake caregivers despite being awake. In agreement with the caregiver-reported age-relevance of AS symptoms, clinical experts reported that anxiety typically worsens in adolescence, whereas disruptive behaviors and seizures tended to improve or be better managed.

### Key Concepts in AS and Those Needing Treatment

When caregivers were asked which concepts were the most challenging for them to manage, decreased expressive communication (n = 11; 37%) and general communication (n = 9; 30%) impairments were among the most frequently reported. Other commonly reported concepts included seizures (n = 7; 27%), sleep (n = 7; 23%), mobility issues (n = 6; 20%) and disruptive behaviors (n = 5; 17%). Caregivers were asked to list aspects of AS that would be the most important target for future treatments for AS. The symptoms that ranked highest were expressive communication (n = 14; 47%), sleep (n = 9; 30%), and seizures (n = 7; 23%). Although multiple concepts relating to AS were discussed in the interviews, when data from clinicians and caregivers are combined, it is evident that there are several key concepts of interest that are frequently reported, are considered challenging, and should be considered as targets for future therapies. The seven concepts where there was convergence of the clinician and caregiver interviews are: seizures; maladaptive behaviors; communication; sleep; motor function; cognition; and self-care (Table 5).

**Table 5:** Most important AS concepts according to clinical experts and caregivers

Concept	Symptoms reported as most challenging, by *clinicians* (n = 3)	Symptoms most frequently reported, by *caregivers* (n ≥ 20)	Symptoms reported as most challenging, by *caregivers* (n = 30)	Most important focus for new treatment, by *caregivers* (n = 30)	Most important focus for new treatment, by *clinicians* (n = 3)
Communication	✓	✓	✓	✓	✓
Seizures	✓	✓	✓	✓	–
Maladaptive behaviors	✓	✓	✓	–	✓
Cognition	✓	✓	–	✓	–
Motor	✓	✓	✓	✓	–
Sleep	✓	✓	✓	✓	✓
Self-care	✓	✓	✓	–	–

## Discussion

AS is a rare, neurodevelopmental condition with a complex, multifaceted clinical presentation that requires life-long medical care. Although no AS-specific treatments are currently available, research efforts in this area have been increasing in recent years. Both clinicians and researchers working with individuals with AS therefore need tools to adequately reflect the complexity of AS and to target efforts for treatment harmonization and coordination across clinical specialties.

From a clinical standpoint, although healthcare systems can differ by geographic and cultural context [10], patients with AS typically require care in multiple specialized settings, as well as regular reviews with their family doctor or pediatrician [26]. It is therefore especially important that all clinicians caring for individuals with AS be aware of their patients’ complex and heterogeneous clinical needs. It is also important that clinicians base their communication with their patients’ families and with other healthcare providers on a common agreement of the specific treatment needs for both the individuals and their caregivers. This goal can only be reached by understanding the differences between the clinicians’ and caregivers' perspectives on AS key concepts, so a focus on open communication and trust remains important in AS care coordination [27].

From a research perspective, an accurate identification of the disease-defining concepts of AS is a prerequisite for the development of appropriate outcome measures for clinical trials to capture meaningful treatment benefit [14]. Importantly, the selection of appropriate outcome measures becomes especially relevant in trials studying neurodevelopmental disorders. This is because the complex and multi-symptomatic nature of these conditions can make defining treatment efficacy particularly challenging, and capturing a potential therapeutic benefit harder to measure. In addition, the use of a structured approach focused on patient-relevant outcomes is recommended by key global health authorities, but can require particular consideration in rare disease drug development [14, 28].

Our research aim was to build a conceptual model that outlines the features of AS and captures its complexity and heterogeneity, from the perspective of the healthcare provider and that of the individuals’ own families. We believe that these results provide valuable insights for both clinical practitioners and researchers to consider alongside the holistic and coordinated management of patients and the development of therapeutic targets.

Our final conceptual model captures seven key disease-defining concepts in AS that are also important treatment targets: seizures, sleep disturbances, limited expressive communication, impaired motor skills, disruptive behaviors, cognition, and limited self-care abilities. Of these concepts, four were unanimously endorsed for their importance by caregivers and clinicians, specifically: seizures, sleep disturbance, communication impairment, and motor difficulties. Clearly, these concepts constitute key clinical features of AS, with impact on both individuals and their caregivers. Clinicians should therefore prioritize symptoms in those areas for treatment. Importantly, these disease-defining concepts span the AS phenotypic categories of consistent, frequent, and associated features, so are not experienced by all individuals with AS [8]. Similarly, researchers should focus on these aspects when selecting appropriate endpoints for clinical trials of new treatments for AS. Eliminating or relieving these symptoms would be expected to provide significant benefit for individuals and their families.

In this instance, the field is fortunate that another conceptual model of AS was recently published by Grieco et al. [29]. Both studies drew from a literature review, followed by clinician and caregiver interviews, but differ in approach. Our work was designed with ongoing input from a steering committee that included clinicians, patient advocates and members of the A-BOM Alliance. Furthermore, our methodological approach represents a broader spectrum of the AS community, including an international perspective, both mother and father caregivers, younger age range, all AS genotypes, extensive clinical expert experience, and comparisons of impacts by age.

By comparing and contrasting the work of Grieco et al. with our data shown here, a fuller understanding of the disease concept of AS can be built on a broader subject base. Although the findings of Greico and colleagues were organized in a different way, it is possible to compare specific concepts and domains across the two studies. Both studies identified concepts in the AS-defining domains of Communication, Neurological, Behavior, Cognition, Motor, Sleep, Musculoskeletal, Gastrointestinal, Skin, Visual and Emotional. Interestingly, the current study elicited concepts at a more granular level, especially in the Communication, Motor, Neurological, and Sleep domains. In terms of the impact of AS on the individual, both studies agree that all activities of daily living are affected, as are social interactions and learning skills. Moreover, both studies agree that the impacts on caregivers are overwhelming, can lead to emotional issues, decreased work productivity, disruptions to family and social life, and reduced sleep. In addition, our work identified a financial burden faced by families of people with AS, due to the need for caregiver assistance as well as the reduced work capacity for parents.

Another important aspect emerging from our research is the different perspective between clinicians and caregivers in assessing importance of some clinical features of AS. Caregivers found that communication impairment in individuals with AS and their inability to take care of themselves were symptoms that were relevant and impactful on personal and family life, but clinicians did not rate these items as among the most important. While the special needs of individuals with AS persist over a lifetime, we found that their relative importance changes from childhood to adolescence. For example, the lack of expressive communication and the need for assistance to use the toilet, bathe or dress themselves may become more challenging as the AS individual grows. Therefore, it is important that clinicians assess each family’s challenges in the context of the individual’s age, and recognize that problems may persist over a lifetime, but their relative importance changes with the age of the individual. This concept is also important for clinical researchers since the efficacy of future AS treatments and the relevance of any treatment on the core symptoms of AS could differ depending on the patient’s age, potentially leading to an altered definition of treatment benefit across the age spectrum.

As previously commented, the selection of AS defining concepts in our conceptual model was guided by the relevance and frequency of those AS features reported in caregivers’ interviews and by the importance rating assigned by both clinicians and caregivers. However, it is worthwhile noting that some of the health issues more frequently reported by families of children with AS are not core features of the syndrome itself, yet represent areas of significant concern for the caregivers. Examples include poor bladder control, fecal incontinence, constipation, reflux, swallowing and feeding problems, and sensitivity to heat. Although these symptoms are not specific to AS, they represent significant challenges for caregivers, and should be recognized by clinicians as they strive to deliver holistic, comprehensive, coordinated and high-quality medical care to patients and their families. Moreover, for both clinicians and researchers, these additional concepts may represent important areas of focus for the development of preventive and therapeutic interventions [30].

Limitations in the methodology of this study include that caregiver respondents may not represent all caregivers of individuals with AS since selection was limited to those who responded to study advertisements via the advocacy organizations. Additionally, half our sample included caregivers of young children aged 5–11 years (n = 14), eight caregivers of adolescents (12–17 years) and only six caregivers of young adults (18–29 years). This sample is, therefore, not representative of older adults with AS. The caregiver sample was also highly educated, which could imply they are better informed and have better access to healthcare resources than the general caregiver population of individuals with AS.

Furthermore, although we incorporated the experience and reports of both families and physicians in the Netherlands and in the USA, these are not necessarily representative of the global community of practitioners and families involved in the care of individuals with AS. Future studies should seek to validate these findings in more diverse samples of experts and families.

Finally, our sample was unbalanced across the two countries of interest (a ratio of 5:1 in favor of USA-based respondents). While this may have led to a focus on concepts elicited from USA-based caregivers, our analyses did not identify any major differences in the sample based on geography, increasing our confidence that these insights are balanced and representative of the two geographies.

## Summary

Our research highlights the impact that AS has on both individuals and caregivers, and compares the perception of AS between clinicians and families. It adds to published literature that provides a comprehensive overview of the life domains affected by AS and illustrates its complex and heterogeneous nature, and the spectrum of challenges it poses for families. Moreover, our work identifies several aspects of AS that are treatment targets that should be considered as domains of interest in AS to develop appropriate, clinically meaningful endpoints in clinical trials. This research can also support harmonized, comprehensive, and coordinated clinical care for AS individuals and their families.
